# Sensitivity and specificity of mean platelet volume as a laboratory marker for irritable bowel syndrome: Can it be added to Rome criteria?

**DOI:** 10.4102/ajlm.v9i1.1001

**Published:** 2020-12-21

**Authors:** Mostafa Vaghari-Tabari, Soheila Moein, Durdi Qujeq, Mehrdad Kashifard, Haydeh Alaoddolehei, Karimollah Hajian-Tilaki

**Affiliations:** 1 Department of Clinical Biochemistry and Laboratory Medicine, Tabriz University of Medical Sciences, Tabriz, Iran; 2 Liver and Gastrointestinal Diseases Research Center, Tabriz University of Medical Sciences, Tabriz, Iran; 3 Molecular Medicine Research Center, Hormozgan University of Medical Sciences, Bandar Abbas, Iran; 4 Department of Biochemistry, Faculty of Medicine, Hormozgan University of Medical Sciences, Bandar Abbas, Iran; 5 Cellular and Molecular Biology Research Center (CMBRC), Health Research Insititute, Babol University of Medical Sciences, Babol, Iran; 6 Department of Clinical Biochemistry, Babol University of Medical Sciences, Babol, Iran; 7 Department of Internal Medicine, Gastroenterology Division, Ayatollah Rouhani Hospital, Babol University of Medical Sciences, Babol, Iran; 8 Department of Hematology and Medical Laboratory Sciences, Para Medical Faculty, Babol University of Medical Sciences, Babol, Iran; 9 Department of Biostatistics and Epidemiology, Babol University of Medical Sciences, Babol, Iran

**Keywords:** IBS, irritable bowel syndrome, inflammatory bowel disease, red blood cell distribution width, mean platelet volume

## Abstract

**Background:**

Irritable bowel syndrome (IBS) is a functional gastrointestinal disorder.

**Objective:**

This study aimed to evaluate red blood cell distribution width (RDW) and mean platelet volume (MPV) as laboratory markers to discriminate IBS patients from both healthy controls and patients with inflammatory bowel disease (IBD).

**Methods:**

This case-control study enrolled patients referred to Ayatollah Rouhani Hospital, Endoscopy Department, Babol, Iran, for colonoscopy examination from 2015 to 2017. Fifty IBS patients were selected from among patients who had undergone a normal colonoscopy and showed symptoms matching the Rome III criteria. Fifty healthy participants and 50 IBD patients, matched for sex and age, were also enrolled in this study. Both RDW and MPV were measured and analysed by independent sample *t*-test and receiver operating characteristic curve analysis. A *p*-value of less than 0.05 was considered statistically significant.

**Results:**

While RDW was higher and MPV was lower among IBS patients compared to healthy controls (*p* = 0.047 and *p* = 0.001), there were no significant differences in RDW or MPV levels between IBS and IBD patients. The area under the curve of RDW in the discrimination between IBS and IBD was 0.620 (*p* = 0.039), and the area under the curve of MPV in the discrimination between healthy controls and IBS patients was 0.801 (*p* = 0.001).

**Conclusion:**

Mean platelet volume is potentially a useful laboratory marker for distinguishing between IBS patients and healthy individuals. Red blood cell distribution width should be considered as a potential marker to distinguish among IBS and IBD patients.

## Introduction

Bowel disorders have been categorised into organic and functional diseases. Organic diseases have observable and measurable disease processes. For example, these diseases may be associated with tissue damage. Inflammatory bowel disease (IBD) and colorectal cancer are examples of important organic intestinal diseases. There are no organic pathologies such as masses and ulcers in intestinal functional disorders.^1^ Irritable bowel syndrome (IBS) is one of the most common functional gastrointestinal disorders associated with abdominal pain and a range of other symptoms like stomach cramps, bloating, diarrhoea, constipation, or alternate periods of diarrhoea and constipation.^2^ The exact pathogenesis of IBS is not clear but it is believed that impairment in the brain-gut axis causes IBS. It seems that multiple factors, including environmental factors such as diet, stress, and intrinsic factors, such as epigenetics and genetics, are involved in IBS pathogenesis and can affect the brain-gut axis.^2^ Irritable bowel syndrome symptoms and severity vary from one individual to another. Irritable bowel syndrome is commonly diagnosed by clinical signs and symptoms according to the Rome III criteria.^3^ Although colonoscopy is not required in the diagnosis of IBS, a colonoscopy can be requested to rule out organic diseases such as IBD in patients with rectal bleeding. Based on the Rome III criteria proposed in 2006, an IBS patient is one who has had recurrent discomfort or abdominal pain 3 days per month in the last 3 months and who has met two or more of the following criteria: decrease in pain or discomfort after defecation, change in stool frequency or change in stool form.^4^ According to signs and symptoms, IBS is divided into four groups, namely: IBS-D—diarrhoea is the predominant symptom; IBS-C—constipation is the predominant symptom; IBS-M—alternating periods of diarrhoea and constipation is a predominant symptom; IBS-U—no predominant symptom is experinced.^5,6^ In addition to the Rome III criteria, a simple laboratory test with acceptable sensitivity and specificity will aid the diagnosis of IBS.

The effectiveness of laboratory tests for IBS diagnosis is poorly investigated. However, some studies have shown that red blood cell distribution width (RDW) and mean platelet volume (MPV) may be useful for IBS diagnosis.^7,8^ Both RDW and MPV are hematologic markers with clinical significance and are routinely included in the complete blood count test. Not only is RDW an indicator of erythrocyte size variation and conventionally used for categorisation of anaemia,^9^ it is also high in some clinical conditions such as autoimmune disease, liver disorder and sickle cell disease.^9,10^ Some studies have demonstrated high RDW in IBD.^11,12^ Meanwhile, MPV has been traditionally used for examination of platelet production status in the bone marrow and has clinical importance in some circumstances^13^; MPV levels may also be altered in hypertension, diabetes and IBD.^14^ Irritable bowel syndrome is an organic intestinal disease with two major subtypes: ulcerative colitis and Crohn’s disease.^15^ Its clinical signs and symptoms have been shown to have a significant overlap with IBS.^5^ Some studies of IBD have demonstrated that MPV can be used for assessment of disease activity.^14,16,17^ Although IBS is conventionally diagnosed based on clinical symptoms, a further laboratory test can be done to increase the validity of the diagnosis. Additionally, patients may need a colonoscopy, for example colonoscopy can be requested for patients who have a family history of IBD. However, this could be avoided if an available laboratory test that accurately discriminates IBS from an organic disorder like IBD. This would also reduce the incidences of colonoscopy. Thus this study evaluated the utility of RDW and MPV as potential laboratory biomarkers to discriminate between IBS and healthy controls as well as for distinguishing among IBS and IBD patients.

## Methods

### Ethical considerations

This retrospective study is a part of Master of Science (MSc) thesis project (no. 6793) and has conformed to the standards of the World Medical Association, as embodied in the Declaration of Helsinki and the protocol was approved by the Hormozgan University of Medical Sciences Ethical Committee (IR.HUMS.REC.94.182). All patients signed the written informed consent forms provided by Hormozgan University of Medical Sciences and agreed that their medical information could be used in this study. These forms contained information about the project and how patient information was used. To protect patients’ personal information, a special code was assigned to each patient’s information.

### Study design and sample selection

All participants were over 18 years of age and were referred to a gastroenterologist at the Ayatollah Rouhani Hospital, Babol in northern Iran. The consultation period was from September 2015 to January 2017.

### Irritable bowel syndrome case group

These were patients whose clinical symptoms matched the Rome III criteria, who had normal C-reactive protein (CRP) and erythrocyte sedimentation rate without organic disorders, confirmed by colonoscopy examination. Patients’ symptoms included abdominal pain, diarrhoea, constipation and other IBS symptoms. Primary reasons for colonoscopy included rectal bleeding, family history of colorectal malignancy and positive stool occult blood test. Colonoscopy examination was done by expert gastroenterologists using an Olympus colonoscopy instrument (Olympus, Tokyo, Japan). Irritable bowel syndrome patients with any of the following criteria were excluded: abnormal haemoglobin level, iron deficiency, abnormal blood smear microscopic analysis, any type of cancer, diverticular disease, history of colorectal surgery, any type of blood disease, diabetes, cardiovascular dysfunction, liver and kidney disease, any type of infectious disease, any type of congenital disease or use of non-steroidal anti-inflammatory drugs (e.g. using aspirin before blood sampling and colonoscopy examination).

A case group of 50 patients, 23 women and 27 men, met the inclusion criteria and were enrolled as IBS patients. Among these IBS patients, 18 patients had IBS-D, 17 patients had IBS-C and 15 patients had IBS-M.

### Inflammatory bowel disease case group

These were patients diagnosed with IBD by colonoscopy and approved by histopathologic methods. Inflammatory bowel disease patients who met the following criteria were excluded: previous colorectal surgery, diverticular disease, cardiovascular disease, cancers, infectious disease, liver diseases, kidney diseases, congenital blood disorders, diabetes and taking non-steroidal anti-inflammatory drugs before blood sampling.

Fifty IBD patients (14 of them had Crohn’s disease and others had ulcerative colitis) were matched for sex and age. Among these IBD patients, 25 patients had active disease and 25 patients were in clinical remission.

### Control group

The control group consisted of 50 healthy volunteers, matched for sex and age, who were referred to the laboratory of Ayatollah Rouhani Hospital, in Babol in northern Iran. The healthy participants were selected after consultation with a gastroenterologist. All did not have a colonoscopy examination, clinical signs and symptoms of IBS or a history of IBD. Healthy individuals were excluded if they had: any systemic condition, any type of anaemia or blood disorder, abnormal haemoglobin, abnormal CRP or erythrocyte sedimentation rate levels, abnormal blood smear, abnormal iron levels or IBS sign or symptoms.

### Laboratory measurements

A venous blood sample was taken from each participant’s arm into ethylenediaminetetraacetic acid-tubes for complete blood count testing. For a precise selection of patients and controls routine laboratory analysis including complete blood count, erythrocyte sedimentation rate, CRP, iron profile and blood smear microscopic analysis was performed for all participants. Red blood cell distribution width and MPV were determined simultaneously with complete blood count in a Sysmex cell counter instrument (Sysmex, Kobe, Japan). The CRP, serum iron and total iron-binding (TIBC) levels were quantitatively measured in an auto-analyser instrument (Hitachi, Tokyo, Japan). The following assay kits were used: Bionic CRP kit (Bionic, Tehran, Iran), serum iron kit (Darman Faraz Kave, Tehran, Iran) and TIBC kit (Darman Faraz Kave, Tehran, Iran). Serum ferritin level was measured by the enzyme-linked immunosorbent assay method according to the ferritin enzyme-linked immunosorbent assay kit instructions (Pishtaz Teb, Tehran, Iran) and using an RT2100c enzyme-linked immunosorbent assay reader instrument (Raytolife, Hamburg, Germany). Erythrocyte sedimentation rate was measured by the conventional Westergren method. In this method, 2 mL of blood sample collected in a tube containing 0.4 mL of sodium citrate was transferred to a standard Westergren-Katz tube. The tube was set to stand for 1 h, for erythrocytes to settle. The column of separated plasma was measured, along with the rate of settling in millimetres per hour.

### Statistical analysis

The data obtained were analysed using an IBM Statistical Package for the Social Sciences (SPSS software version 17, Armonk, New York, United States). The independent sample *t*-test was used for comparing variable means between groups. The receiver operating characteristic analysis was also used for comparing the utility of MPV and RDW for discrimination between IBS patients and healthy controls as well as for discrimination between IBS and IBD patients. *P*-values less than 0.05 were considered as statistically significant.

## Results

Our data demonstrated that the MPV level was significantly reduced while the RDW level was significantly elevated in IBS patients when compared with those of healthy subjects, but the levels of the same parameters were not significantly different between IBS and IBD patients (Table 1).

**TABLE 1:** Demographic and laboratory characteristics of patient and control groups, Babol, Iran, 2015–2017.

Variables	IBS patients	IBD patients	Healthy controls	*P*-value comparison between IBS patients and healthy controls	*P*-value comparison between IBS patients and IBD patients
Age	34.00 ± 9.93	34.00 ± 10.20	36.00 ± 10.34	0.553	0.663
RDW % ± SD	13.09 ± 1.70	13.78 ± 2.02	12.56 ± 0.69	0.047	0.068
MPV fL ± SD	9.24 ± 0.80	9.23 ± 0.96	10.11 ± 0.74	0.001	0.964

MPV, mean platelet volume; RDW, red cell distribution width; IBS, irritable bowel syndrome; IBD, inflammatory bowel disease; fL, femtoliter; SD, standard deviation.

**FIGURE 1:**
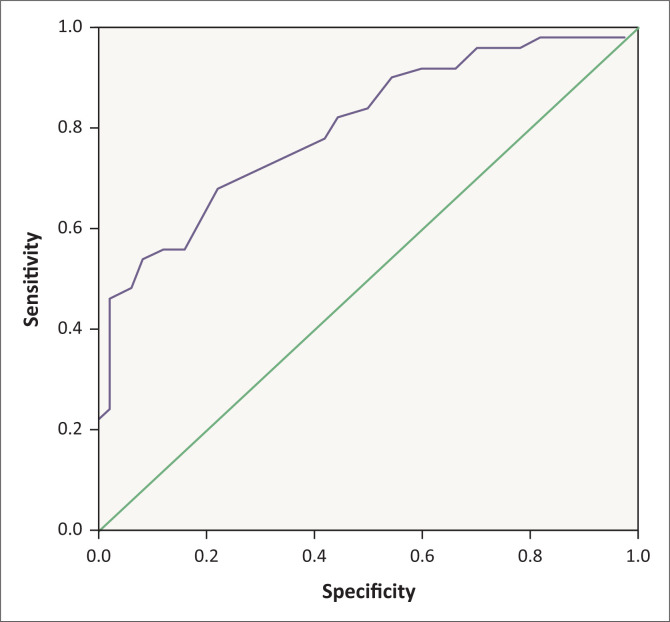
Receiver operating characteristic curve analysis to evaluate mean platelet volume utility in discriminating between healthy controls and irritable bowel syndrome patients, Babol, Iran, 2015–2017.

**FIGURE 2:**
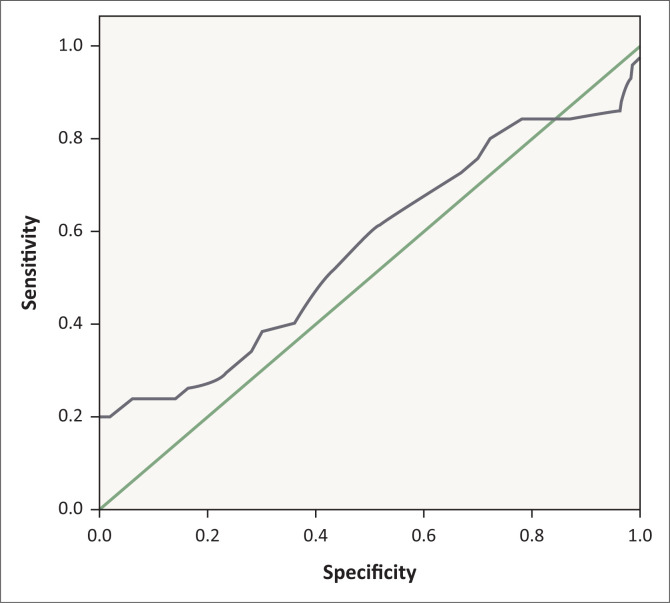
Receiver operating characteristic curve analysis to evaluate red blood cell distribution width utility in discriminating between irritable bowel syndrome patients and healthy controls, Babol, Iran, 2015–2017.

Receiver operating characteristic analysis showed that MPV has a larger area under the curve (0. 801, *p* = 0.001) than RDW (Figure 1, Figure 2, Table 2) in differentiating between the healthy control and IBS patients. Mean platelet volume had a 68% sensitivity and 88% specificity at 9.55 femtoliter.

**TABLE 2:** Receiver operating characteristic curves for comparison of mean platelet volume and red blood cell distribution width utility in discriminating between healthy controls and IBS patients, Babol, Iran, 2015–2017.

Markers	Area	Standard error	Asymptotic significance	Asymptotic 95% confidence interval	
				Lower bound	Upper bound
Mean platelet volume	0.801	0.044	0.000	0.716	0.887
Red blood cell distribution width	0.564	0.058	0.270	0.450	0.678

**FIGURE 3:**
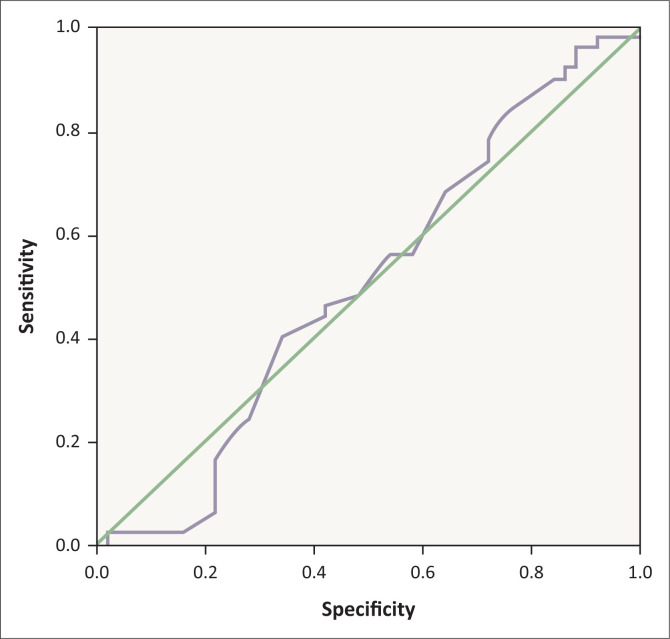
Receiver operating characteristic curve analysis to evaluate mean platelet volume utility in discriminating between irritable bowel syndrome and inflammatory bowel disease patients, Babol, Iran, 2015–2017.

**FIGURE 4:**
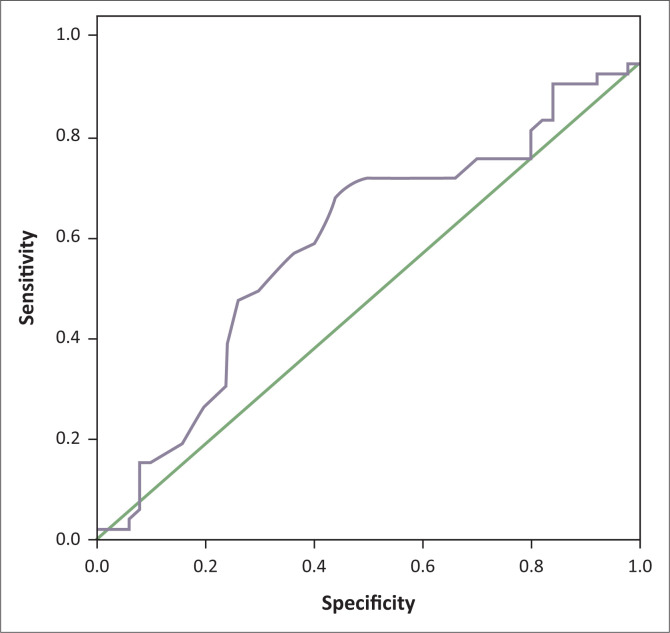
Receiver operating characteristic curve analysis to evaluate red blood cell distribution width utility in discriminating between inflammatory bowel disease and irritable bowel syndrome patients, Babol, Iran 2015–2017.

Receiver operating characteristic analysis showed that RDW compared to MPV had a larger but not significant area under the curve of 0.620 (*p* = 0.039) for differentiating between IBS and IBD patients (Figure 3, Figure 4, Table 3). The best cut-off point for RDW was a value of 13.05%, with 72% sensitivity and 56% specificity, to help discriminate IBD patients from IBS patients.

**TABLE 3:** Receiver operating characteristic curves for comparison of mean platelet volume and red blood cell distribution width utility in discriminating between irritable bowel syndrome and inflammatory bowel disease patients, Babol, Iran, 2015–2017.

Markers	Area	Standard error	Asymptotic significance	Asymptotic 95% confidence interval	
				Lower bound	Upper bound
Mean platelet volume	0.502	0.059	0.975	0.387	0.617
Red blood cell distribution width	0.620	0.057	0.039	0.508	0.731

## Discussion

Our results showed that there was not a significant difference in RDW and MPV between IBS and IBD patients. Although average RDW among IBS patients was lower compared with RDW among IBD patients, the difference was not statistically significant (*p* = 0.068). However, this difference could become meaningful if the sample size were increased. Our data also showed that, in comparison with healthy controls, the mean MPV was lower while RDW was higher among IBS patients, which could be due to subclinical inflammation in IBS patients. Subclinical inflammation in IBS has been previously reported in some studies,^18,19^ and it is well known that MPV and RDW can be altered in inflammatory conditions.^11,12,14^ Our findings augment the hypothesis that subclinical inflammation has a role to play in IBS. However, further studies in this regard are needed. Our findings, in contrast to a study conducted in Turkey, showed that the MPV levels were higher among IBS patients than among healthy controls.^7^ This inconsistency may be due to the difference in study populations or inclusion criteria. In our study, the results of receiver operating characteristic curve analysis showed that the area under the curve, sensitivity and specificity of MPV for IBS diagnosis are acceptable. The use of receiver operating characteristic curve analysis to evaluate the utility of MPV in IBS diagnosis has not been previously presented by other studies; thus, a direct comparison of our results with other studies, results was not possible. Inflammatory bowel disease is a complex gastrointestinal disease and multiple factors are involved in its pathogenesis.^20,21,22^ According to our results, RDW has a relatively low specificity for differentiating between IBD and IBS patients. Our results are similar to a study conducted in Hungary that reported 92% specificity and 78% sensitivity for RDW (cut-off: 13.4%) in active Crohn’s disease diagnosis. These researchers also reported that in the majority of IBS patients RDW was normal. They however did not evaluate the specificity and sensitivity of RDW in discriminating between IBD and IBS.^23^

Further studies are needed to evaluate the utility of RDW as a diagnostic marker. There are very few studies investigating laboratory diagnosis of IBS and it is proposed that the MPV may be used as an IBS biomarker. A mean platelet volume test in addition to the clinical parameters of the Rome criteria can improve the diagnosis of IBS.

### Limitations

Our study has some limitations. Firstly, a major study limitation is that our sample size was relatively low because the aim of the study was primarily to evaluate the utility of RDW and MPV for laboratory diagnosis of IBS. Budget and time limitations further limited the possible sample size. The second limitation of our study was that we could not assess the other laboratory factors to discriminate between healthy controls and IBS patients. However, we used rigorous inclusion and exclusion criteria for patient selection in an attempt to mitigate the influence of confounding conditions.

### Conclusion

Red blood cell distribution width was higher while the MPV level was lower among IBS patients compared to healthy controls, although the same parameters did not differ significantly when compared with IBD patients. Mean platelet volume is a potential marker with adequate sensitivity and specificity to discriminate healthy controls from IBS patients. However, it is not useful for discriminating between IBD and IBS patients. Red blood cell distribution width could be a potential marker for differentiation between IBD and IBS. Further studies with a sufficiently large sample size are needed for a comprehensive evaluation of the utility of these potential biomarkers to discriminate between healthy controls, IBS patients and IBD patients.
